# Notes and News

**Published:** 1892-01-30

**Authors:** 


### Notes and News.

Collected and Communicated.

A correspondence has been going on in the *Glasgow Press* with reference to a proposed hospital for poor consumptives to be situated a few miles out of the town. Dr. Charles Workman, Dr. Hugh Thomson, and other medical men are in favour of the scheme.

The Committee have placed a small stained-glass window in the day-room of the Norwood Cottage Hospital to the memory of the late Mr. John Sharman, M.R.C.S., he having for eight years been one of the hon. surgeons of the above hospital. The subject of the window is "The Good Samaritan."

It is a far cry to Coldstream, yet the little Cottage Hospital there, which was started by the Countess of Home, is an excellent institution, and worthy of note. During last year fifty-one patients were treated, including five typhoid and five scarlet fever cases. This little hospital also has one bed set aside for incurable cases. The income was £290, and the expenditure £261. District nursing is also carried on by the Matron and nurses.

Aberdeen City Hospital has been the latest victim of the scandal craze, and the Town Council had the wisdom to immediately appoint a sub-committee to investigate the charges. The result was that the scandal was reduced to the fact that one of the servants read one of the patient's letters —a regrettable fact, but still no ground for the wholesale abuse of nurses, and the tales of wailing children which had been circulated. In future the patients are to see their letters locked into the cage which will be put in the disinfecting chamber. We congratulate all concerned on the prompt manner in which this scandal was met and squashed.

In the appeal issued for help towards the renovation and enlargement of the Radcliffe Infirmary, Oxford, attention is drawn to a criticism contained in "The Hospital Annual" for the present year. This criticism seems to have impressed the authorities, as they now pass it on in their circular to the inhabitants of the city and county of Oxford, who, we hope, will be aroused to a sense of their position, and speedily subscribe so modest a sum as £10,000. At any rate, the action now taken by the Committee of Management absolves them for the present from further responsibility in the matter, whatever the result of the appeal may be. Should it fail, and we do not think it possible that it can fail, Oxford will certainly have to submit to take a back seat in the hospital world as well as in the world of humanity and culture.

An important meeting of the representatives of the London Hospitals and those interested in their welfare will be held at the Society of Arts, John Street, Adelphi, on Thursday, February 4th, at 8.30 p.m. The chair will be taken by the Right Hon. the Lord High Chancellor, and Mr. B. Burford Rawlings will deliver an address on "The Chronic Indigence of our Hospitals." Several representative speakers have promised to attend; tickets may be obtained on application to the Secretary of the Hospitals Association, 140, Strand, W.C., and it is hoped that all who are interested in hospitals will make a special point of being present. No one is better fitted by long experience and intimate knowledge to deal effectively with the needs of our hospitals than Mr. Rawlings, and we hope that the Press and the public may be induced to take a warm interest in the meeting, and to combine to make it the means of securing a largely increased measure of support for all Metropolitan medical charities.

This is an age when the majority indulge in reading. Unfortunately, the reading is for the most part of a very useless description, and so ends at best in a waste of time. There are a class of people who would positively prevent children from reading fairy tales, on the ground that they appeal too strongly to the imagination of young minds. Such people are forgetful of the fact, as an able critic remarks, that all our best writers have been born and bred on fairy stories, and no child ever yet suffered a pang of terror from perusing them. No doubt it is a great mistake to allow children to spend all their leisure in reading. Such a practice makes them positively silly, it numbs all their faculties, prevents the natural development of the muscles, and deprives them of necessary exercise and air, without which a child becomes a pigmy, although some foolish people may regard it as a prodigy. It is of the first importance to encourage the cultivation of the minds of children by the reading of wholesome and amusing literature. It is also of the first importance that the hours of leisure shall be so apportioned, as to permit of an adequate amount of athletic exercise with a due regard to the development of the muscles, the expansion of the lungs, and the general health of a child. These are simple truths, but unfortunately many people seem quite unable to act upon them so far as their own children are concerned.

At a time when deaths from pneumonia and bronchial disease are alarmingly on the increase it is well to direct attention to the great need which exists for more hospital accommodation for consumptive cases all over the country, and especially in London. A movement is now on foot to establish in Dublin a National Hospital for Consumption for Ireland, of which Miss Florence Wynne, 115, Lower Baggot Street, Dublin, is the Honorary Secretary. Of the existing institutions, owing to the great pressure on their beds, the majority refuse admission to advanced cases of consumption, and confine their relief mainly, if not entirely, to patients who are likely to receive benefit from hospital treatment. Having regard to the phthisical tendency of large numbers of the population owing to the climateric conditions of these islands we are strongly of opinion that an urgent effort should be made to extend the North London Consumption Hospital at Hampstead to its fullest capacity. Further, it is desirable that one or more additional hospitals for consumption should be organised on a wide basis in suitable localities, so as to secure that all who need hospital treatment may have a reasonable hope of obtaining it before it is too late to be of any real benefit. Wealthy philanthropists and others casting about for an opening for the wise expenditure of a good round sum as a memorial, or from sheer kindness of heart, should enquire into this matter of consumption hospitals, and see that the inadequacy of the present provision is speedily supplemented. Cannot the scare of the present season due to the ravages of influenza be utilised to secure the necessary funds to enable adequate hospital provision to be made for the relief and care of consumptive cases throughout Great Britain?

